# Mapping Knowledge Landscapes and Emerging Trends of the Links Between Frailty and Heart Failure: A Bibliometric Analysis From 2000 to 2023

**DOI:** 10.7759/cureus.60511

**Published:** 2024-05-17

**Authors:** Ning Zhang, Xuan Qu, Lin Kang, Xiaohong Liu, Wenling Zhu

**Affiliations:** 1 Department of Geriatrics, Peking Union Medical College Hospital, Peking Union Medical College, Beijing, CHN; 2 Department of Cardiology, Peking Union Medical College Hospital, Peking Union Medical College, Beijing, CHN

**Keywords:** quantitative analyses, citespace, vosviewer, bibliometrics, heart failure, frailty

## Abstract

Background

Frailty, within the context of heart failure (HF), is strongly linked to poor patient outcomes. Investigating the vulnerable condition of individuals with HF is crucial, not only for medical reasons but also as a significant public health challenge, especially among the elderly population where both HF and frailty are common. Therefore, it is essential to prioritize HF patients with frailty over those without such symptoms. To begin, promptly assessing the impact of academic research in this area is crucial, considering factors such as geographical regions, authors, journals, and institutions. Additionally, it is important to explore current topics and identify potential areas that could inspire future researchers to conduct further studies to advance public health.

Methodology

We conducted a search in the Web of Science Core Collection database to identify articles and reviews in the English language focusing on frailty and HF which were published from January 1, 2000, to December 31, 2023. To perform bibliometric analysis, VOSviewer (v.1.6.18) and CiteSpace (v.6.1.R2) were utilized.

Results

A total of 1,381 original English-language articles were gathered, comprising 1,162 articles and 219 reviews. The quantity of research publications in this area has experienced significant growth since 2013. Among all countries, the United States has contributed the largest number of publications, accounting for 409 articles (29.62% of the total). Additionally, the United States has received the highest number of citations, being cited a total of 13,329 times, as well as boasting the greatest total link strength. Duke University stands out as the institution with the highest number of research papers, having published 40 articles (2.90% of the total). It has also received the most citations, with a total of 2,455 times, and possesses the highest total link strength, which amounts to 212. Within the realm of prolific authors, Kentaro Kamiya from Kitasato University emerges as the most productive, having authored 28 articles (2.03% of the total). When considering scholarly journals, “Esc Heart Failure” contains the highest number of articles pertaining to frailty and HF, publishing a noteworthy 36 articles (2.61% of the total). Noteworthy keywords within this field encompass frailty, heart failure, elderly, mortality, and cardiovascular disease. Over the past five years, the most popular keywords have centered around “frailty syndrome,” “sarcopenia,” and “therapeutic interventions.”

Conclusions

Research on frailty and HF at a global scale has experienced substantial growth between 2000 and 2023, demonstrating a prospective field for further exploration with potential advantages from ongoing progress. Prospective studies could prioritize the enhancement of cardiac rehabilitation for patients coping with HF and frailty while ensuring the preservation of their overall quality of life.

## Introduction

Frailty is a condition characterized by decreased physiological capacity in multiple organ systems, resulting in heightened susceptibility to functional decline and negative health outcomes following exposure to a stressor that disrupts homeostasis [1]. Various tools and criteria are utilized to assess frailty in individuals. The Cardiovascular Health Study (CHS) criteria and the Frailty Index are commonly used measures for diagnosing frailty. In addition to these, other tools such as the FRAIL (fatigue, resistance, ambulation, illness, and loss of weight) Scale, Edmonton Frail Scale, Tilburg Frailty Indicator, Kihon Checklist, and the five-item frailty screening index have been demonstrated to be valid assessments for determining frailty in individuals [2]. Depending on the chosen definition, the prevalence of frailty among individuals aged 65 years or older with cardiovascular disease (CVD) varies between 15% and 63% [3]. Patients diagnosed with CVD and frailty face an elevated risk of unfavorable clinical outcomes and treatment-related adverse events. Consequently, frailty is increasingly taken into consideration when making treatment decisions, especially when the intervention is associated with significant physiological stress (e.g., surgery) or the potential for serious adverse events (e.g., falls) [4].

Frailty, in the context of heart failure (HF), has a strong association with patient outcomes [5]. It is prevalent among individuals diagnosed with heart failure with reduced ejection fraction (HFrEF) and is linked to decreased exercise capacity, reduced quality of life, and an increased risk of adverse cardiovascular events [3,6]. Its estimated prevalence in HF patients is 44.5%, making it a crucial factor in determining outcomes within this population [7]. A systematic review examining HF studies demonstrated that frailty was associated with a 48% higher risk of death (hazard ratio (HR) = 1.48, 95% confidence interval (CI) = 1.31-1.65) and a 40% higher risk of HF hospitalization (HR = 1.40, 95% CI = 1.27-1.54) compared to non-frail individuals [8]. To investigate the relationship between frailty and death/hospitalization outcomes in HF patients, a prospective cohort study was conducted [9]. This study included a total of 3,429 adults with HF from 27 countries, representing different income levels. The participants had a mean age of 61 ± 14 years, and baseline frailty was assessed using the Fried index. The study revealed that 18% of participants were categorized as robust, 61% as pre-frail, and 21% as frail at baseline. The participants were monitored for a median follow-up period of 3.1 years. Cox proportional hazard models were employed to analyze the data, adjusting for various factors, such as country income level, age, sex, education, and clinical variables. The results demonstrated that pre-frail and frail individuals had significantly higher HRs for death compared to robust participants. Additionally, the HRs for hospitalization due to HF were also greater among pre-frail and frail participants. These findings remained consistent across different income levels and subgroups. Overall, the study suggested that frailty plays a crucial role in predicting death and hospitalization outcomes in HF, and this relationship holds true across countries with varying income levels. A recent retrospective cohort study examined 38,843 newly admitted HF patients to evaluate frailty using the multidimensional frailty index. The study employed Cox regression models and Fine and Gray subdistribution hazard models to assess the impact of frailty on clinical outcomes over one and two years of follow-up. Findings revealed that among the older HF patients (mean age = 80.4 ± 8.5 years, 52.3% females) studied, 68.3% were classified as frail, with 47.5% displaying mild frailty and 20.8% severe frailty. The median number of readmissions increased with the severity of frailty (fit: 1 [interquartile range (IQR) = 2], mild frailty: 1 [IQR = 2], severe frailty: 2 [IQR = 3]). Overall, 27.3% of HF patients, regardless of frailty status, succumbed to CVD. In comparison to the fit group, the severe frailty group exhibited a higher risk of all-cause mortality (adjusted HR = 1.16, 95% CI = 1.11-1.21), all-cause readmissions [subdistributional hazard ratio (sHR) = 1.21, 95% CI = 1.16-1.25], and HF-related readmissions (sHR = 1.14, 95% CI = 1.09-1.20) at the two-year follow-up. The study concluded that frailty is highly prevalent among older HF patients and is associated with an increased risk of all-cause mortality, all-cause readmissions, and HF readmissions [10].

Given these findings, it is imperative to prioritize the investigation of the vulnerable condition of individuals with HF. This is not only a matter of medical importance but also a significant public health obstacle, particularly among the elderly demographic where both HF and frailty are prevalent. Hence, it is crucial to give more attention to HF patients with frailty in comparison to those who do not exhibit such symptoms. To commence, it is essential to promptly assess the impact of academic research in this field, taking into consideration factors such as geographical regions, authors, journals, and institutions. Furthermore, it is vital to explore current subjects and identify potential matters that can inspire future researchers to conduct additional studies to advance public health. In this study, VOSviewer and CiteSpace are utilized to quantitatively scrutinize the pertinent literature concerning the investigation of frailty and HF from 2000 to 2023. The knowledge acquired from this analysis will contribute to novel research perspectives in the coming years.

## Materials and methods

Ethics

The study was reviewed and approved by the Ethics Committee of Peking Union Medical College Hospital (approval number: I-23PJ738).

Data sources and search strategies

To conduct our research, we accessed the primary data source, the Extended Science Citation Index in the Web of Science Core Collection (WoSCC). The WoSCC database is well-known for its extensive coverage of scientific research, encompassing more than 21,000 peer-reviewed journals across diverse disciplines, including science, social science, and humanities [11]. Renowned for its comprehensive bibliometric analysis, this database provides invaluable details such as abstracts, titles, author keywords, author names, affiliations, country/region information, references, and citation analysis. Given its distinguished reputation, numerous scholars regard it as the preeminent option for bibliometric studies and have extensively utilized it in previous investigations [12].

**Figure 1:**
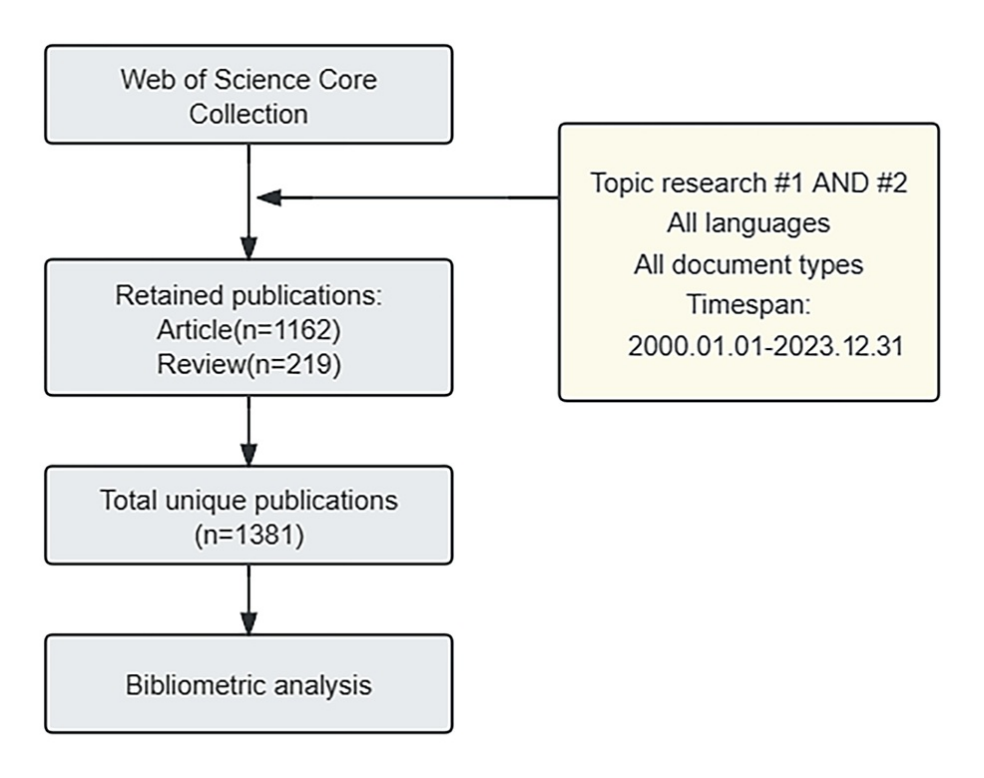
The flowchart of study identification and selection showing the detailed literature selection and screening process. Topic search #1(“Heart Failure” OR “Cardiac Failure” OR “Heart Decompensation” OR “Myocardial Failure”); Topic search #2(“Frailty” OR “Frailties” OR “Frailness” OR “Debility” OR “Debilities”).

The Web of Science is a widely recognized database for bibliometric analysis due to its comprehensive coverage across various disciplines, meticulous citation indexing, and diverse analysis indicators. These features enable researchers to identify research trends and key topics in their respective fields. For this study, we collected publication data on frailty and HF from the WoSCC database for bibliometric analysis. All searches, data extraction, and downloads were performed on the same day to mitigate any potential bias from data updates. To ensure impartiality, we limited the scope of the WoSCC database to include only English-language publications published between January 1, 2000, and December 31, 2023. We specifically focused on original articles and reviews, excluding duplicate articles, editorials, letters, and conference abstracts. The search strategy used in this study was TS = (“Heart Failure” OR “Cardiac Failure” OR “Heart Decompensation” OR “Myocardial Failure”) AND TS = (“Frailty” OR “Frailties” OR “Frailness” OR “Debility” OR “Debilities”).. A total of 1,381 original English-language articles underwent screening, comprising 1,162 articles and 219 reviews. The details of the literature selection and screening process are outlined in Figure 1.

Data collection and bibliometric analysis

As part of our analysis, we collected data on all the literature included. The data obtained was converted into a plain text format for further examination. We then extracted bibliometric measures, including the yearly count of publications and citations, as well as identified significant contributors such as authors and their respective countries/regions.

To conduct a more in-depth examination and representation of the bibliometric data, we used the powerful tools CiteSpace software and VOSviewer software. VOSviewer was effectively employed to generate a comprehensive overview of collaborations on a national and institutional level, as well as to analyze keyword co-occurrences. In contrast, the advanced features of CiteSpace 6.1.R2 were employed to visualize and analyze highly cited articles, literature expansion, and various other aspects.

The node size in the network maps created by CiteSpace indicates the number of publications or citations. CiteSpace offers two metrics, modularity Q (Q value) and mean silhouette (S value), to assess the consistency of the network structure and clustering in the co-citation reference cluster map. A Q value exceeding 0.3 suggests a notable cluster structure, while an S value surpassing 0.7 denotes strong clustering. Total link strength (TLS) is employed for gauging the intensity of connections between various subjects and determining their placement within the collaborative or co-citation correlation.

Research tools and methods

VOSviewer, developed in 2009 by van Eck and Waltman at Leiden University’s Center for Science and Technology Studies in the Netherlands, is a software that primarily focuses on visually representing scientific knowledge using literature data. It utilizes a similarity matrix and VOS mapping technology to cluster data effectively. For our analysis, we used VOSviewer version 1.6.18 to visualize country cooperation, institutional cooperation, and keyword distribution.

CiteSpace, a constantly updated information visualization tool created by Prof. Chaomei Chen, employs Java for the analysis and visualization of the evolutionary trajectory of emerging themes through knowledge graphs [13]. In our study, we employed CiteSpace to visualize and analyze highly cited literature, identify emerging trends, and conduct burst analysis on keywords. Additionally, CiteSpace enables the overlay of bi-images on journals [14].

## Results

Global trends in the publications

**Figure 2:**
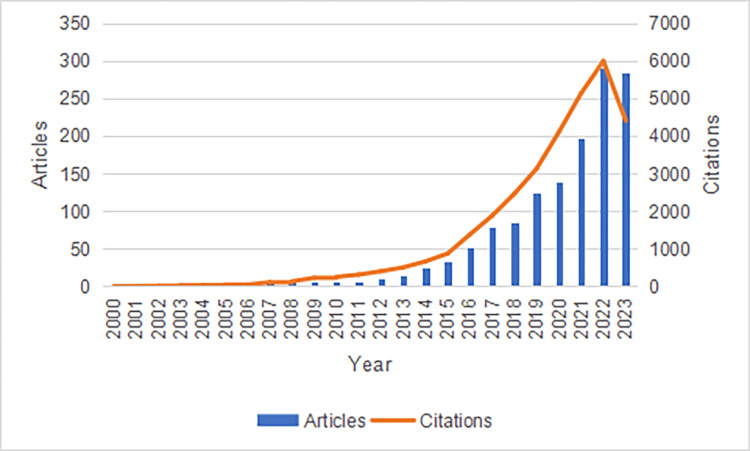
The annual publication volume and annual citation frequency of relevant articles from 2000 to 2023 are displayed on the timeline, with the horizontal axis representing time. The main vertical axis on the left shows the yearly publication counts, while the secondary axis on the right shows the yearly citation frequencies.

Figure 2 presents the annual number of publications and the yearly citation frequency of articles related to frailty and HF from 2000 to 2023. It shows an upward trend in both annual publications and citation frequency. The citation frequency experienced a notable increase after 2019. In 2022, the number of annual publications and citation frequency reached their highest point, with 291 publications and 6,004 citations.

Analysis of countries and regions

In total, 75 countries and regions have published literature on HF combined with frailty. To evaluate a country or region’s participation in this field, we surveyed to determine the number of articles published by each country or region. Based on the data presented in Table 1, it is evident that the United States has the highest number of articles (409), followed by Japan (184) and Italy (124), all of which have published over 100 articles. The United States also has the highest citation frequency (13,329), accounting for approximately 46% of the total citations. Other countries such as Canada (4,059), Italy (2,436), Japan (1,892), and the United Kingdom (1,676) also have a relatively high citation frequency.

**Table 1:** Top 10 countries in terms of number of publications, total link strength, and the frequency of citations.

Rank	Countries	Publications	Countries	Total link strength	Countries	Citations
1	United States	409	United States	268	United States	13,329
2	Japan	184	Italy	188	Canada	4,059
3	Italy	124	England	166	Italy	2,436
4	Canada	99	Germany	155	Japan	1,892
5	England	87	Canada	123	England	1,676
6	China	72	Poland	114	Germany	1,421
7	Australia	66	Sweden	112	Spain	1,408
8	Spain	63	Scotland	110	Australia	1,190
9	Germany	57	Spain	107	The Netherlands	881
10	Poland	44	Denmark	105	Switzerland	843

**Figure 3:**
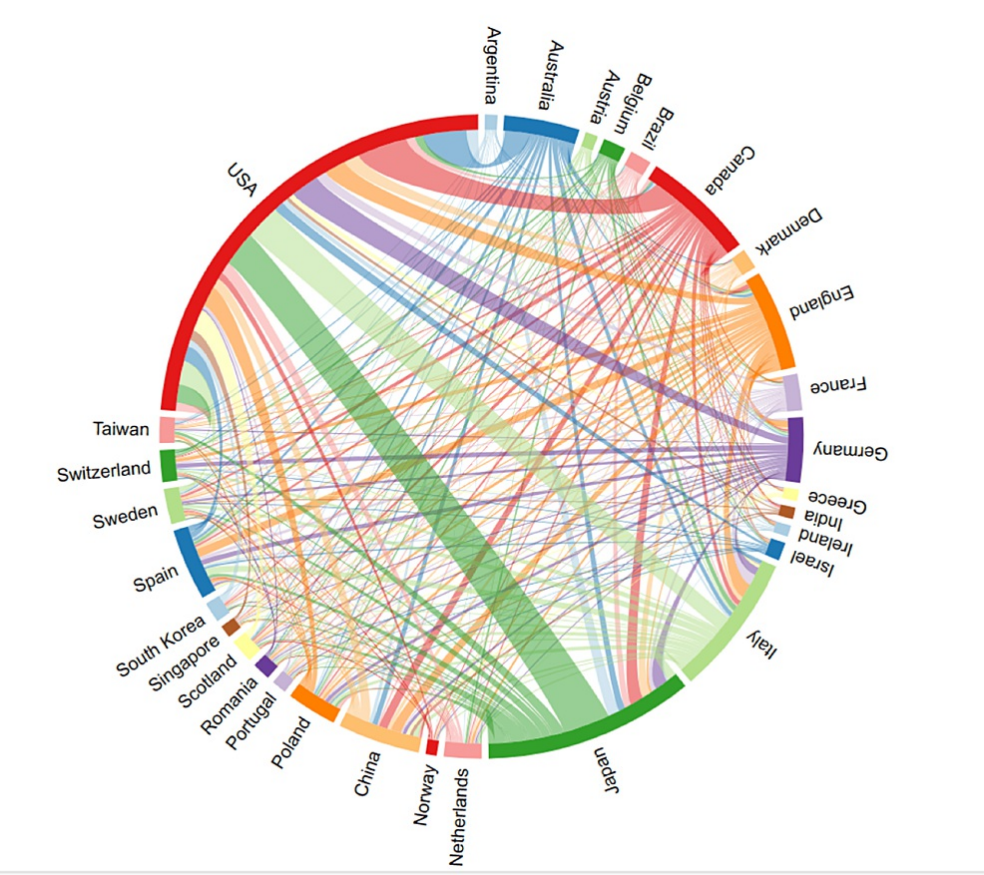
Countries and regions clustering analysis was conducted using collaboration information data sourced from the Web of Science Core Collection (WoSCC) and visualized using Charticulator.

The top three countries with the highest TLS are the United States, Italy, and the United Kingdom. A chord diagram in Figure 3 visualizes and analyzes the cooperation relationships between countries or regions. The size of the circles represents the percentage of articles published in each country or region, while the thickness of the connecting lines reflects the strength of the partnership. Among all countries/regions, the United States has established the most extensive cooperative relationships, particularly with nations such as Italy and Japan. The United States has made a significant contribution to the research of chronic HF in combination with frailty, surpassing the efforts of other countries. Currently, the United States occupies a dominant position in the discourse and leads the field in this area.

Analysis of the authors and co-cited authors

Co-cited author analysis refers to the simultaneous citation of documents by two authors, as done by a third author. The frequency of co-citation indicates the proximity of academic interests and research density [15]. By examining the authors with the highest number of publications and co-citations in the study of HF associated with frailty, we can gain insights into the research strength of these authors and the research hotspots related to this topic. Table 2 presents the top 10 authors based on publication volume and co-citation frequency. The authors with the most published articles are Kentaro Kamiya (28), followed by Emi Maekawa (25), Dalane W. Kitzman (20), and Peter S. Macdonald (20). Among these authors, Kentaro Kamiya (341) and Emi Maekawa (334) have a high TLS, indicating their close cooperative relationships with other authors. Linda P. Fried (560) is the most cited author, followed by Jonathan Afilalo (373) and Kenneth Rockwood (358). Additionally, Linda P. Fried has the highest TLS of 6,021, ranking first, followed by Jonathan Afilalo (4,620) and Kenneth Rockwood (4,257).

**Table 2:** Top 10 authors and co-cited authors related to heart failure with frailty.

Rank	Author	Documents	Total link strength	Author	co-citations	Total link strength
1	Kentaro Kamiya	28	341	Linda P. Fried	560	6,021
2	Emi Maekawa	25	334	Jonathan Afilalo	373	4,620
3	Dalane W. Kitzman	20	91	Kenneth Rockwood	358	4,275
4	Peter S. Macdonald	20	66	John E. Morley	268	3,272
5	Nobuaki Hamazaki	19	220	Piotr Ponikowski	163	1,541
6	Kohei Nozaki	19	220	Sunita R. Jha	161	2,261
7	Izabella Uchmanowicz	17	13	Quin E. Denfeld	159	1,980
8	Junya Ako	16	140	Ambarish Pandey	155	1,783
9	Yuya Matsue	16	247	Jack M. Guralnik	138	1,522
10	Masaaki Konishi	15	221	Stefan D. Anker	134	1,543

**Figure 4:**
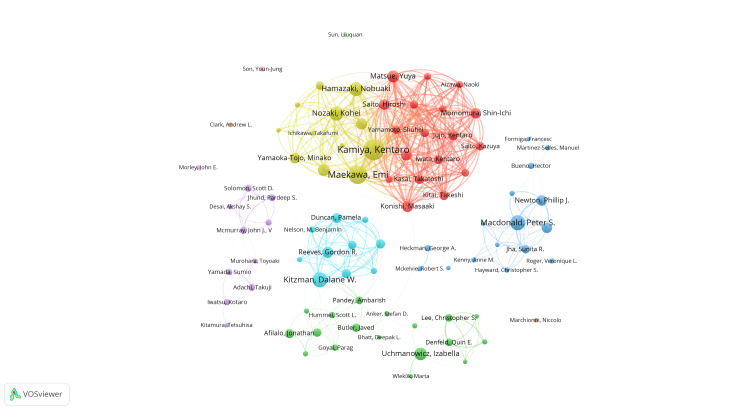
Author analysis. Co-authors in the field of frailty and heart failure. The author of each circle represents a different author. The circle line reflects the connection between the author, and different colors of the network represent the author groups often cooperating. All author information data in the figure are sourced from the Web of Science Core Collection (WoSCC) and visualized using VOSviewer.

**Figure 5:**
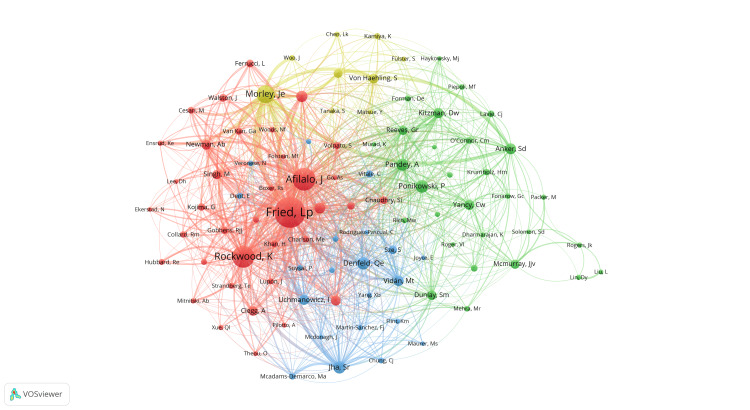
Author analysis. Co-cited-author map in the field of frailty and heart failure. The same color indicates that nodes are in the same cluster. Different colored nodes represent the authors of different collaborative relationships. Word size, circle size, and connection thickness are positively correlated with co-citation frequency. All author information data in the figure are sourced from the Web of Science Core Collection (WoSCC) and visualized using VOSviewer.

Figure 4 and Figure 5 depict a network of authors, with each circle representing a different author and the lines indicating their connections. The various colored clusters highlight groups of authors who frequently collaborate. In Figure 4, the clustering analysis of co-authors reveals that cooperation among scholars in this field is scattered and requires closer and more extensive connections. By analyzing co-referenced author relationships, we find that the yellow network, involving Kentaro Kamiya, and the red network, comprising Yuya Matsue, Masaaki Konishi, and others, are the most comprehensive and centrally positioned. This suggests that these authors have extensive and active collaborations with others. Figure 5 presents a network diagram illustrating the relationships between the authors’ research on HF combined with frailty. The diagram is divided into four blocks. The red block includes authors such as Linda P. Fried and Kenneth Rockwood. The green block includes authors such as Piotr Ponikowski and Ambarish Pandey. The blue block includes authors such as Izabella Uchmanowicz and Sunita R. Jha. The yellow block includes authors such as John E. Morley, and so on.

Contributions of institutions

The information presented in Table 3 highlights the top 10 institutions based on several factors, including publication quantity, frequency of collaboration with other institutions, and citation frequency. According to the publication ranking, Duke University (United States) has the highest number of published papers, with 40 papers. Brigham & Women’s Hospital (United States) (34) and Kitasato University (Japan) (32) closely follow with slightly fewer articles published by other institutions. Duke University (United States) is the most frequently cited institution, with 2,455 citations, followed by the University of Pittsburgh (United States) (1,681), McGill University (Canada) (1,630), Brigham & Women’s Hospital (United States) (1,606), and Johns Hopkins University (United States) (1,600). The remaining institutions have been cited fewer than 1,600 times. It is noteworthy that Japan’s institutions dominate the top 10 in terms of TLS, indicating a strong and collaborative relationship among Japan’s institutions in this field.

**Table 3:** Top 10 institutions in terms of number of articles issued, total link strength, and the frequency of citations.

Rank	Institution	Publications	Institution	Total link strength	Institution	Citations
1	Duke Univ	40	Kitasato Univ	212	Duke Univ	2,455
2	Brigham & Women’s Hosp	34	Juntendo Univ	197	Univ Pittsburgh	1,681
3	Kitasato Univ	32	Yokohama City Univ	190	Mcgill Univ	1,630
4	Mayo Clin	27	Kameda Med Ctr	185	Brigham & Women’s Hosp	1,606
5	Juntendo Univ	26	Jichi Med Univ	184	Johns Hopkins Univ	1,600
6	Univ Pittsburgh	26	Nishiarai Heart Ctr Hosp	183	Univ Colorado	1,407
7	Johns Hopkins Univ	24	Saitama Citizens Med Ctr	182	NIA	1,331
8	Wroclaw Med Univ	23	Sakakibara Heart Inst Okayama	182	Columbia Univ	1,185
9	Thomas Jefferson Univ	22	Odawara Municipal Hosp	174	Mayo Clin	974
10	Jichi Med Univ	20	Shinshu Univ Hosp	169	Wake Forest Sch Med	950

**Figure 6:**
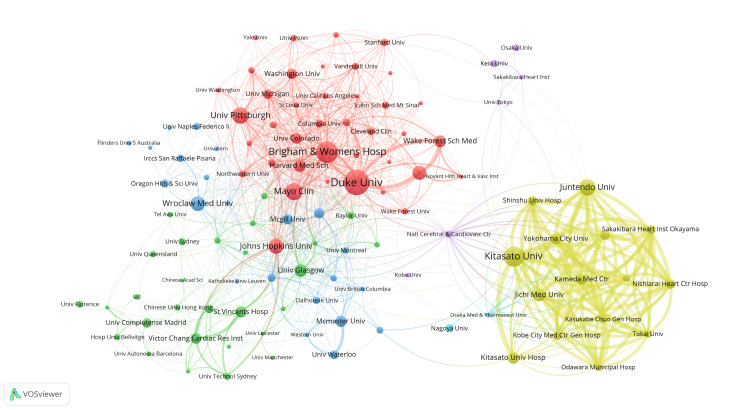
Institution analysis. Visualization of collaborative network among institutions using VOSviewer. Nodes of varying colors represent different clusters of institutions, and the node size corresponds to the frequency of their occurrence. All institution collaboration information data in the figure are sourced from the Web of Science Core Collection (WoSCC) and visualized using VOSviewer.

Our research involved conducting a cluster analysis of published institutions to gain insights into the global distribution of research on frailty and HF. The aim was to provide reference data for cooperation between institutions. Figure 6 displays the clustering analysis of the institutions involved. The red and yellow clusters represent more extensive cooperative institutional networks. The red cluster includes institutions such as Brigham & Women’s Hospital, Duke University, and the University of Pittsburgh. The yellow cluster showcases cooperation with Japanese institutions, including Kitasato University and Juntendo University, among others. However, we identified a need for a greater connection between these two clusters. The dark blue cluster includes institutions such as Wroclaw Medical University and McGill University. The green clusters include the University of Glasgow and St. Vincent Hospital. The purple and light blue clusters have relatively fewer institutions.

**Figure 7:**
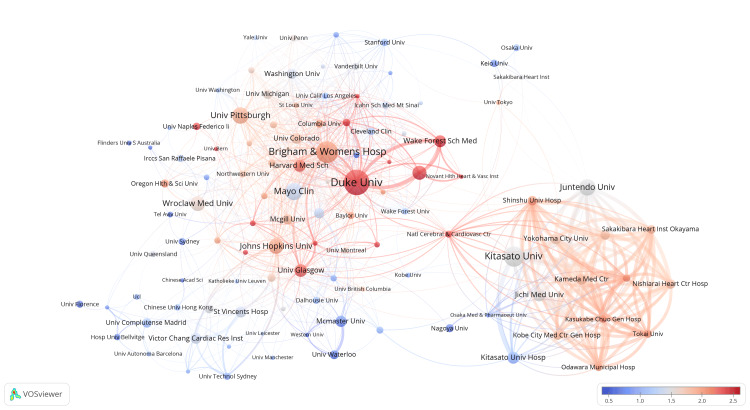
Institution analysis. The proportion of institutional publications to complete publications during the past few years. All institution collaboration information data in the figure are sourced from the Web of Science Core Collection (WoSCC) and visualized using VOSviewer.

We used VOSviewer to analyze the data of articles published in recent years. As shown in Figure 7, McMaster University, Stanford University, and Kitasato University conducted research in this field earlier and have published more articles in the past than in recent years. On the contrary, institutions such as Duke University conducted research relatively late. The cluster analysis graph shows that the red areas mainly consist of institutions from the United States and Japan, indicating that they entered the field rather late or have recently published many articles.

Analysis of journals and co-cited academic journals

**Figure 8:**
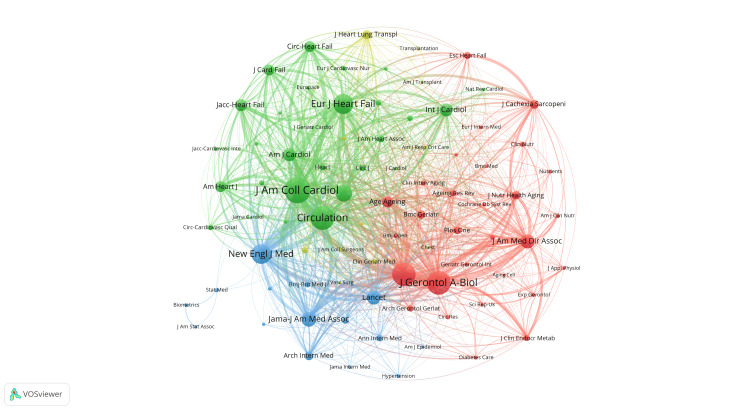
Visualization of journals’ citation networks using VOSviewer. The size of the nodes reflects the frequency of their occurrence. All journal citation information data in the figure are sourced from the Web of Science Core Collection (WoSCC) and visualized using VOSviewer.

We utilized an online bibliometric analysis platform to identify top journals with high publication rates and significant impact in our field of study. As shown in Table 4, Esc Heart Failure (3.8, Q2) (36) had the highest number of publications, followed by the Journal of the American Geriatrics Society (6.3, Q1) (31) and Journal of Clinical Medicine (3.9, Q2) (23). The most co-cited journal was J Am Coll Cardiol (24.4, Q1) (1,950), followed by J Gerontol A-Biol (5.1, Q2) (1,687) and Circulation (37.8, Q1) (1,681). Figure 8 illustrates the relationship between the journals that published articles on frailty and HF. These journals are categorized into four blocks based on similarity, represented by different colors. The red block primarily includes J Gerontol A-Biol, J Am Med Dir Assoc, and J Nutr Health Aging. The green block mainly consists of journals such as J Am Coll Cardiol, Circulation, Eur J Heart Fail, etc. The blue block includes prominent journals such as New England J Med, The Lancet, JAMA, etc. The yellow block comprises specific journals such as J Heart Lung Cancer and periodicals such as J Heart Lung Transpl, J Am Coll Surg, and Clin Genet. The red block of journals focuses on research areas in geriatrics and geriatric health (nutrition and aging, clinical practice in geriatrics, etc.). The serials in the yellow block are more focused on clinical practice, research, and the use of genetics in CVD and surgery (cardiac surgery, heart-lung transplantation, immunosuppressive therapy, etc.). The journals in the green block concentrate on cardiovascular medicine (HF and heart transplantation, etc.). In contrast, the journals in the blue area encompass top global medical journals with high academic impact and authority, covering a wide range of research areas without a specific focus.

**Table 4:** Top 10 journals and co-cited journals related to frailty and heart failure. JCR = Journal Citation Reports; IF = impact factor

Rank	Journal	Publications	IF (JCR 2022)	JCR quartile	Co-cited-journal	Citations	IF (JCR 2022)	JCR quartile
1	Esc Heart Failure	36	3.8	Q2	J Am Coll Cardiol	1,950	24.4	Q1
2	Journal of the American Geriatrics Society	31	6.3	Q1	J Gerontol A-Biol	1,687	5.1	Q2
3	Journal of Clinical Medicine	23	3.9	Q2	Circulation	1,681	37.8	Q1
4	JACC-Heart Failure	22	13	Q1	J Am Geriatr Soc	1,647	6.3	Q1
5	Journal of Cardiac Failure	21	6.7	Q1	Eur J Heart Fail	1,340	18.2	Q1
6	American Journal of Cardiology	18	2.8	Q3	New Engl J Med	1,291	158.5	Q1
7	Circulation Journal	18	3.3	Q2	Eur Heart J	1,046	39.3	Q1
8	International Journal of Cardiology	18	3.5	Q2	Jama-J Am Med Assoc	874	120.7	Q1
9	Frontiers in Cardiovascular Medicine	16	3.6	Q2	J Am Med Dir Assoc	825	7.6	Q1
10	European Journal of Heart Failure	15	18.2	Q1	Lancet	795	168.9	Q1

Analysis of keywords

The primary focus of the article is the combination of HF and frailty. The forefront of research in this area has been analyzed using keywords. Table 5 presents the top 20 keywords with the highest frequency of occurrence. The most commonly used keywords are “framework” (462), “heart failure” (378), and “elderly” (213). Other frequently used keywords include “mortality” (93), “cardiovascular disease” (65), “sarcopenia” (61), and “aging” (60). These keywords highlight the significance of these topics in the field of HF with frailty.

**Table 5:** Top 20 keywords in terms of frequency of occurrence and the corresponding total link strength.

Rank	Keyword	Occurrences	Total link strength	Rank	Keyword	Occurrences	Total link strength
1	Frailty	462	990	11	Atrial fibrillation	28	70
2	Heart failure	378	779	12	Geriatrics	28	70
3	Elderly	213	494	13	Comorbidity	27	73
4	Mortality	93	240	14	Malnutrition	26	62
5	Cardiovascular disease	65	145	15	Quality of life	25	68
6	Sarcopenia	61	155	16	Hospitalization	23	71
7	Aging	60	134	17	Readmission	22	59
8	Prognosis	52	134	18	Chronic heart failure	21	43
9	Outcomes	38	77	19	Cognitive impairment	21	69
10	Cardiac rehabilitation	31	70	20	Risk factors	21	45

**Figure 9:**
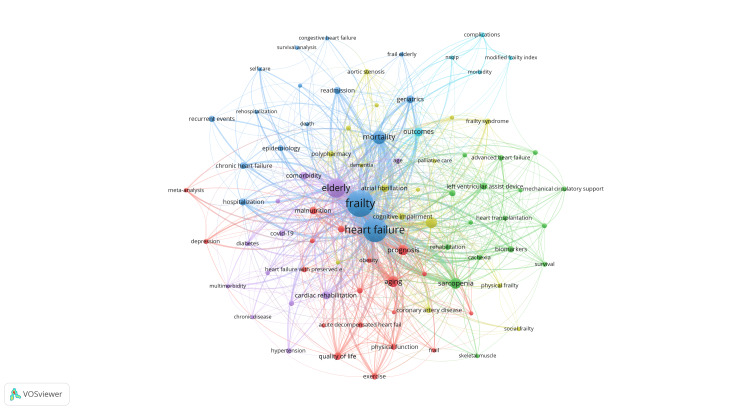
Analysis of frailty and heart failure keywords. Visualization of keyword collaboration network using VOSviewer. Nodes of varying colors represent different clusters of keywords, and the node size corresponds to their frequency. All keyword information data in the figure are sourced from the Web of Science Core Collection (WoSCC) and visualized using VOSviewer.

Keywords with close co-occurrence relationships are clustered into one category, mainly four larger blocks represented by different colors, as shown in Figure 9. The red block primarily relates to HF combined with frailty and mortality (fragility, mortality, HF). The green block mainly relates to the treatments (sarcopenia, biomarkers, left ventricular assist device). The purple block primarily relates to older adults and complications (elderly, comorbidity, cardiovascular). The keywords in the blue block are mainly associated with prognosis (diagnosis, aging, malnutrition).

**Figure 10:**
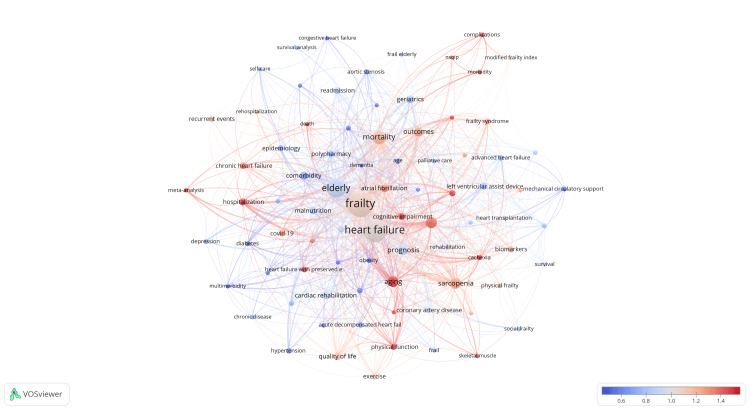
Analysis of frailty and heart failure keywords. The blue/red scale shown in the bottom right corner displays the proportion of the frequency of appearance of the keywords to the total frequency of appearance during the past few years. All keyword information data in the figure are sourced from the Web of Science Core Collection (WoSCC) and visualized using VOSviewer.

It is important to note that the blue blocks are extensively connected to other blocks, indicating their intersection in various related research fields. Figure 10 presents an analysis of the popularity of keywords, where red nodes indicate high popularity and blue nodes represent low popularity. The findings indicate that keywords such as aging, cognitive impairment, and hospitalization have gained increasing popularity. On the other hand, keywords such as comorbidity, diabetes, and obesity have relatively decreased in popularity.

**Figure 11:**
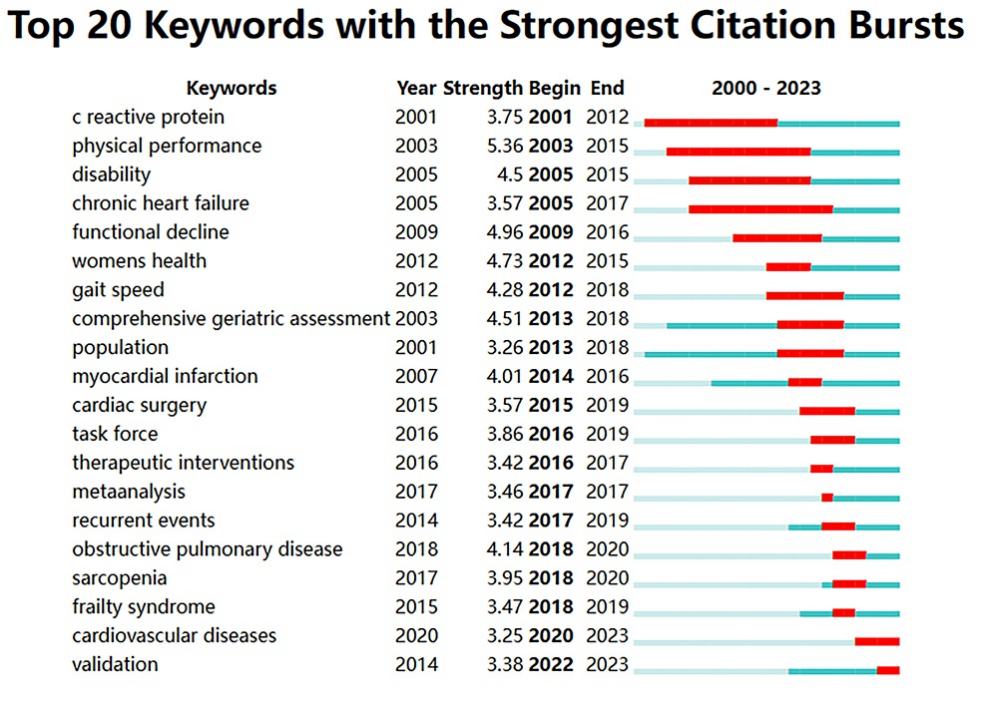
Analysis of frailty and heart failure keywords. CiteSpace visualization map of the top 20 keywords with the strongest citation bursts involved in frailty and heart failure. The blue bars mean the reference has been published, and the red bars represent citation burstness. All keyword information data in the figure are sourced from the Web of Science Core Collection (WoSCC) and visualized using CiteSpace.

In research, keywords with frequent citations indicate hotspots and emerging trends. Figure 11 presents the first 20 keywords in chronological order. The keywords “Physical performance” had the highest citation burst (5.36), followed by “functional decline” (4.96) and “women’s health” (4.73). The graph reveals that the most popular keywords in the past five years mainly revolve around “frailty syndrome” (3.47), “sarcopenia” (3.95), and “therapeutic interventions” (4.14). However, “cardiovascular diseases” (3.25) and “validation” (3.38) continue to be in the spotlight.

Analysis of highly cited references

Table 6 presents the 15 most cited articles, with one article cited over 800 times and five articles cited more than 400 times. It is worth noting that six out of the 15 articles were published in 2010 or earlier. Among these, the paper titled “Intensive vs Standard Blood Pressure Control and Cardiovascular Disease Outcomes in Adults Aged ≥75 Years: A Randomized Clinical Trial” by Williamson et al., published in the Journal of the American Medical Association in 2016, stands out as the most cited paper with a total of 816 citations [16]. The study enrolled individuals aged 75 years or older who were randomly divided into two groups: the group receiving intensive treatment (n = 1,317) with a target systolic blood pressure (SBP) below 120 mmHg, and the group receiving standard treatment (n = 1,319) with a target SBP below 140 mmHg. Frailty was evaluated using the frailty index, and it was determined that 30.9% of participants in this trial were classified as frail at the beginning. Among the participants, 33.4% in the intensive treatment group and 28.4% in the standard treatment group were categorized as frail. The results, when analyzed based on initial frailty status, displayed increased event rates with advancing frailty in both treatment groups. Nevertheless, within each frailty category, the absolute event rates were reduced for the intensive treatment group (p = 0.84 for interaction). These findings demonstrate that the benefits of blood pressure treatment in reducing CVD events and mortality persist regardless of frailty status. Significantly, the study provides evidence that treating elderly individuals aged 75 years or more with an SBP target below 120 mmHg significantly lowers the incidence of fatal and non-fatal major cardiovascular events and overall mortality compared to treating them with an SBP target below 140 mmHg.

**Table 6:** Top 15 articles in terms of frequency of citation.

Rank	Article title	Source title	Cited	Year	Document type	DOI	Author
1	Intensive vs standard blood pressure control and cardiovascular disease outcomes in adults aged ≥75 years: a randomized clinical trial	Journal of the American Medical Association	816	2016	Article	10.1001/jama.2016.7050	Williamson et al. [16]
2	Frailty assessment in the cardiovascular care of older adults	Journal of the American College of Cardiology	692	2014	Article	10.1016/j.jacc.2013.09.070	Afilalo et al. [17]
3	Associations of subclinical cardiovascular disease with frailty	Journals of Gerontology Series A-Biological Sciences and Medical Sciences	585	2001	Article	10.1093/gerona/56.3.M158	Newman et al. [18]
4	Role of frailty in patients with cardiovascular disease	American Journal of Cardiology	420	2009	Article	10.1016/j.amjcard.2009.01.375	Afilalo et al. [19]
5	Sarcopenia and mortality risk in frail older persons aged 80 years and older: results from ilSIRENTE study	Age and Ageing	416	2013	Article	10.1093/ageing/afs194	Landi et al. [20]
6	Hospital readmissions as a measure of quality of health care -: advantages and limitations	Archives of Internal Medicine	400	2000	Review	10.1001/archinte.160.8.1074	Benbassat et al. [21]
7	Freedom from recurrent ventricular tachycardia after catheter ablation is associated with improved survival in patients with structural heart disease: an International VT Ablation Center Collaborative Group study	Heart Rhythm	327	2015	Article	10.1016/j.hrthm.2015.05.036	Tung et al. [22]
8	Profiles of older medicare decedents	Journal of the American Geriatrics Society	252	2002	Article	10.1046/j.1532-5415.2002.50268.x	Lunney et al. [23]
9	Frailty predicts long-term mortality in elderly subjects with chronic heart failure	European Journal of Clinical Investigation	223	2005	Article	10.1111/j.1365-2362.2005.01572.x	Cacciatore et al. [24]
10	Advanced (Stage D) heart failure: a statement from the Heart Failure Society of America Guidelines Committee	Journal of Cardiac Failure	222	2015	Article	10.1016/j.cardfail.2015.04.013	Fang et al. [25]
11	Quality of end-of-life care provided to patients with different serious illnesses	JAMA Internal Medicine	220	2016	Article	10.1001/jamainternmed.2016.1200	Wachterman et al. [26]
12	Interleukin-6 and C-reactive protein, successful aging, and mortality: the PolSenior study	Immunity & Ageing	217	2016	Article	10.1186/s12979-016-0076-x	Puzianowska-Kuznicka et al. [27]
13	Is inappropriate medication use a major cause of adverse drug reactions in the elderly?	British Journal of Clinical Pharmacology	204	2007	Article	10.1111/j.1365-2125.2006.02831.x	Laroche et al. [28]
14	The prevalence of frailty in heart failure: a systematic review and meta-analysis	International Journal of Cardiology	199	2017	Review	10.1016/j.ijcard.2017.01.153	Denfeld et al. [29]
15	Prevalence and prognostic impact of frailty and its components in non-dependent elderly patients with heart failure	European Journal of Heart Failure	198	2016	Article	10.1002/ejhf.518	Vidán et al. [30]

The article “Frailty Assessment in the Cardiovascular Care of Older Adults” by Afilalo et al. (2014) is the second most cited article, with 692 citations. The importance of frailty in guiding cardiovascular care as a therapeutic goal is highlighted in this study. It proposes that future research should integrate frailty to a greater extent to enhance patient prognosis. The researchers suggest that using the recommended assessment tools can be beneficial, as they provide a dependable structure for comparing and combining findings from various studies and evaluating novel indicators of frailty [17]. In 2001, a research study titled “Associations of frailty with subclinical cardiovascular disease” was conducted by Newman et al. The findings of this study suggest that individuals who suffer from CVD are more susceptible to experiencing frail health. Even individuals without a prior history of CVD are at risk of frailty if they have underlying CVD, which can be measured through carotid ultrasound and ankle-arm index, left ventricular hypertrophy by ECG, and echocardiography. The study also discovered a correlation between frailty and infarct-like lesions in the brain, which can be identified using MRI [18].

**Figure 12:**
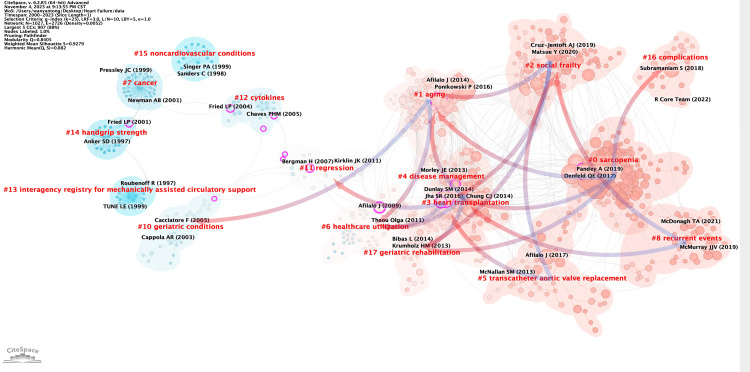
Domain references’ keywords map. The same color indicates that these keywords are in the same clusters, and the links indicate the co-occurrence relationship between the keywords. All information data in the figure are sourced from the Web of Science Core Collection (WoSCC) and visualized using CiteSpace.

Figure 12 presents the clustered relational mapping of citations in this study. The category with the highest number of publications is sarcopenia (#0), followed by aging (#1), social frailty (#2), and heart transplantation (#3). In terms of the time dimension, the earliest research topics in this field are handgrip strength (#14) and interagency registry for mechanically assisted circulatory support (#13). These articles were published in 1997 by Anker et al. and Roubenoff et al. Additionally, cancer (#7), noncardiovascular conditions (#15), geriatric conditions (#10), and cytokines (#12) were also early clusters of interest in this field, with a small number of cross-cited articles. The emerging high co-citation topics are recurrent events (#8), complications (#16), and social frailty (#2), which are closely related. Authors of highly cited articles in these areas are collaborating more, indicating that research on these topics is currently receiving significant attention in the field.

**Figure 13:**
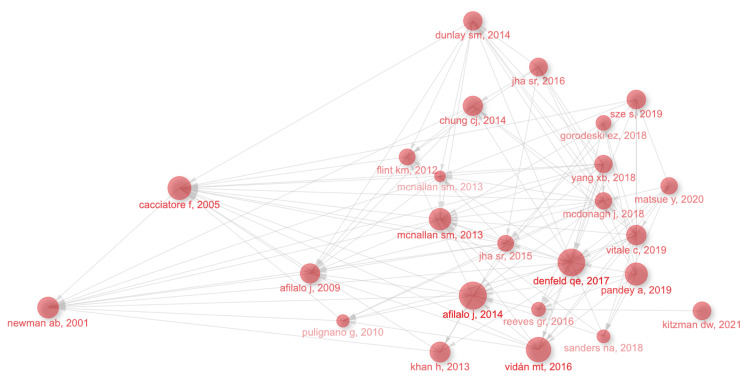
Analysis of references. Relationship graph of highly cited articles. The connecting lines represent the citations between them. Sizes of nodes refer to citations of documents. All reference information data in the figure are sourced from the Web of Science Core Collection (WoSCC) and visualized using “bibliometrix.”

Figure 13 provides a clearer illustration of the developmental structure by offering a more concise analysis of highly cited references. The earliest highly cited articles and articles located at the core nodes are Brach JS’s (2001) “Gait Variability in Community-dwelling Old Adults” [31] and Afilalo et al.’s (2014) “Frailty Assessment in the Cardiovascular Care of Old Adults” [17]. In recent years, Mentz’s (2021) “Rehabilitation Intervention in Older Patients With Acute Heart Failure With Preserved Versus Reduced Ejection Fraction” suggests that customized rehabilitation improves physical functioning more than usual care in the elderly population hospitalized for acute HF [32]. Additionally, Denfeld et al. (2017) published a paper titled “The prevalence of frailty in heart failure: a systematic review and meta-analysis,” where they indicated that frailty is a common problem affecting almost half of the patients with HF, and it is not always related to age or functional classification. Further research should focus on standardizing how frailty is measured and expanding the definition of frailty beyond being solely a geriatric syndrome in HF [29].

**Figure 14:**
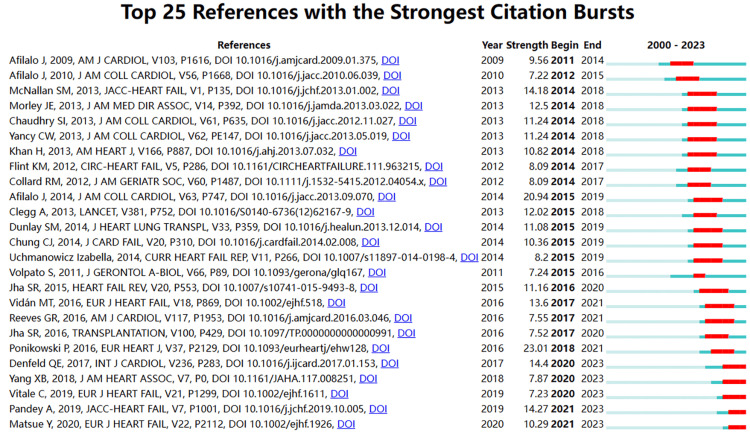
Analysis of references. The top 25 references involving the strongest citation burst in frailty and heart failure, sorted by year of origin; the blue bars mean the reference had been published, and the red bars represent citation burstness. All reference information data in the figure are sourced from the Web of Science Core Collection (WoSCC) and visualized using CiteSpace.

Figure 14 presents the top 25 references with the strongest citation burst. From 2014 to 2018, there were annual outbreaks of citations, lasting approximately four years. Another citation outbreak occurred in 2020, following 2018. Between 2014 and 2015, there were seven and six citation outbreaks, respectively, which continued until 2018 and even 2019, representing the highest level of citation outbreaks in recent years.

## Discussion

HF is a significant public health problem impacting millions of patients and imposing substantial economic strains on countries globally. According to recent findings from the China Hypertension Survey (CHS), conducted from 2012 to 2015, it was revealed that HF affected approximately 1.3% of Chinese adults aged ≥35 years. Consequently, this amounts to an estimated 8.9 million HF patients in the country [33]. Frailty in individuals diagnosed with HF is acknowledged as a reversible and fluctuating condition marked by augmented susceptibility to stressors in clinical and non-clinical domains. This heightened vulnerability leads to adverse consequences and an unfavorable prognosis, giving rise to escalated morbidity, healthcare usage, dependence, and an elevated likelihood of death [34]. The prevalence of frailty in patients with HF is considerable, as frailty is experienced by about 90% of patients with preserved ejection fraction and 30-60% of patients with reduced ejection fraction who have HF. Moreover, there is a 26% higher prevalence of frailty among women compared to men. Importantly, it should be noted that the relationship between frailty and HF is reciprocal, as both conditions can exacerbate one another [35].

In this study, we examined the publishing trends related to the research of frailty and HF and analyzed the countries/regions, journals, and authors that contributed to this emerging field. The number of articles has shown a steady increase from 9 in 2001 to 291 in 2022 and stands at 284 articles as of December 31, 2023. Since 2013, global trends have consistently risen. In terms of published articles, China occupies the sixth position globally. However, it falls short of being among the top 10 countries regarding citations and TLS. In comparison to countries such as the United States, Japan, and Italy, a significant disparity in this domain persists. As a result, it becomes imperative for China to delve into extensive research to tackle the comprehensive intervention and management of patients with HF, particularly in light of the rapid aging trend prevalent in the nation.

Keywords with close co-occurrence relationships can be divided into four main clusters. The first cluster focuses on the mortality of HF combined with frailty, including fragility, mortality, and HF. The second cluster mainly relates to treatments associated with HF, such as sarcopenia, biomarkers, and left ventricular assist devices. The third cluster primarily pertains to older individuals and their coexisting conditions, such as comorbidity and cardiovascular issues. The fourth cluster mainly associates with prognosis, including diagnosis, aging, and malnutrition.

By deeply engaging with extensively referenced articles in this particular domain, individuals can obtain a broader and more multifaceted comprehension of the present-day research situation, trending areas of investigation, recent progressions, the challenges faced in research, and the forthcoming avenues for further exploration. The initial breakthrough in this field occurred in 2011, as demonstrated by the publication of the article “Role of Frailty in Patients With Cardiovascular Disease” by Afilalo in 2009 [19]. In this article, a comprehensive review of studies on frailty in patients with CVD was conducted. This breakthrough continued until 2014. Presently, the reference heavily relies on the recent review by Pandey et al. published in 2019 [6]. This review delves into exploring the burden of frailty and the underlying concepts of frailty in older patients with HF. It also discusses potential strategies for assessing, screening, and managing frailty in this vulnerable population. Furthermore, ongoing research is being conducted to investigate the prevalence, overlap, and prognostic significance of physical and social frailties, as well as cognitive impairment, among elderly HF patients who are admitted to the hospital [36]. This indicates that researchers have begun to recognize that frailty in patients not only affects their physical reserve function, but is also closely linked to factors such as weak social support, the impact of chronic diseases, depression, and cognitive decline. As a result, it is essential for future research to assess patients with HF in a comprehensive manner, taking into account various factors including physical function, cognitive status, psychology, polypharmacy, and social support. This is particularly important for elderly patients with HF, as the Comprehensive Geriatric Assessment can promptly identify and address reversible factors, leading to an improvement in both their quality of life and overall condition.

Timeline view analysis and citation burst detection methods were used to identify cutting-edge content and reveal future trends. From Figure 7 and Figure 9, we found that frailty syndrome, sarcopenia, cardiac rehabilitation, prognosis/outcome, and quality of life are the hotspots of research in recent years. Muscle wasting, as defined using the criteria of sarcopenia, was shown to be a common comorbidity of HF, decreasing the functional capacity of affected patients with either HFrEF or heart failure with preserved ejection fraction (HFpEF) [37,38]. Frailty is further complicated by a higher prevalence of sarcopenia in HF patients compared with patients without HF, which negatively affects outcomes. While both sarcopenia and frailty share similar risk factors (e.g., malnutrition, hormonal changes, and physical activity) and coexist with each other, they are considered distinct entities. Frailty is a syndrome that affects multiple systems and entails a progressive decline in both physical and physiological states. On the other hand, sarcopenia is precisely characterized as the reduction of muscle mass and/or functionality, which contributes to physical frailty [39,40].

Frailty is characterized by significant physical and functional limitations, which can be improved with ongoing aerobic exercise and physical rehabilitation. Consequently, frail individuals with HF can experience improved clinical outcomes through these interventions [41]. The primary focus of the REHAB-HF trial was to evaluate the efficacy of a well-structured program for physical rehabilitation in enhancing recovery among 349 elderly patients diagnosed with HF. All participants in this study were ambulatory and had functional independence before enrollment. This intervention aimed to improve strength, balance, mobility, and endurance among the participants. This rehabilitation program was initiated during hospitalization and was continued post-discharge. The average age of the patients in this study was 73 ± 8 years, with 97% considered frail based on the modified Fried criteria. Following three months, the intervention group reported a significant enhancement in their SPPB score (mean difference compared to the control group, 1.5; 95% CI = 0.9-2.0; p < 0.001). These results continued to remain statistically significant even after adjusting for comorbidities such as diabetes and peripheral vascular disease. Furthermore, notable improvements were observed in six-minute walk testing, frailty status, Kansas City Cardiomyopathy Questionnaire (KCCQ)-Clinical Summary Score, and depression within the intervention group [42]. Pandey et al. analyzed the HF-ACTION trial retrospectively to evaluate how supervised aerobic exercise training affects the efficiency of treatment in individuals with differing levels of frailty. The study consisted of 2,130 participants who were divided into two subgroups based on their level of frailty using the deficit accumulation index. These participants were then followed for a median period of 2.9 years. The results showed a notable decrease in the primary composite outcome in frail patients who underwent exercise training (HR = 0.83, 95% CI = 0.72-0.95), primarily due to a reduction in hospitalizations for all causes. However, there was no significant reduction observed in non-frail patients (HR = 1.04, 95% CI = 0.87-1.25) [43]. In Japan, a retrospective study was conducted across multiple centers to investigate the effects of cardiac rehabilitation on 3,277 ambulatory individuals diagnosed with HF. The findings of the study unveiled a significant reduction in both the occurrence of all-cause mortality and HF admissions in patients who engaged in outpatient cardiac rehabilitation as opposed to those who did not undergo any rehabilitation. This impact was consistently observed in patients diagnosed with HFpEF or exhibiting frailty [44]. These data strongly indicate that cardiac rehabilitation plays a critical role in enhancing the functional outcomes reported by frail individuals suffering from HF.

To examine the quality of life among a diverse population of HF patients, a comprehensive study was conducted in which 23,291 individuals from 40 different countries across eight distinct world regions were included. The aim of the Global Congestive Heart Failure Study (G-CHF) was to evaluate the impact of HF on patients’ well-being. Utilizing the KCCQ-12, the researchers assessed the quality of life of these individuals. To establish a clear connection between the KCCQ-12-SS and the combined outcome of all-cause death, HF hospitalization, and each individual component, a multivariable Cox regression technique was employed. The analysis accounted for 15 relevant variables that could potentially affect the results. Over 1.6 years, the participants in the study were closely monitored, providing valuable longitudinal data. The findings of this investigation demonstrated a consistent and statistically significant relationship between health-related quality of life (HRQoL) and the occurrence of all-cause death and HF hospitalization across all geographic regions. This association persisted regardless of the severity of HF symptoms, ranging from mild to severe, as well as in individuals with both preserved and reduced ejection fraction[45]. Consequently, evaluating the HRQoL will hold significance in describing individuals with HF, aside from other clinical indicators. The forthcoming research emphasis will center on enhancing the quality of life of HF patients, specifically those experiencing frailty, via methodical evaluation.

In general, the significance of frailty in HF has consistently captivated considerable interest. Presently, no universally acknowledged diagnostic criteria exist for frailty. In the future, a standardized evaluation of frailty is required to amplify the applicability of frailty scores and the effectiveness of treatment specifically for frail individuals. The initiative to enhance the quality of care provided to HF patients commences with comprehending the balance between benefits and risks associated with treatments, especially as the patient’s physiological capacity alters with the progression of age. Based on the comprehensive geriatric assessment, the patient’s geriatric syndromes, such as physical frailty, cognitive frailty, and social frailty, are promptly identified, along with the identification and correction of reversible factors. The Geriatric Interdisciplinary Team then provides individualized comprehensive intervention measures, including rehabilitation, which should be the primary focus of future care for frail elderly HF patients. 

There are several limitations associated with this investigation. The study heavily relies on the WoSCC database, known for its vast literary resources and reliable information. However, this could lead to a potentially incomplete literature search. To enhance coverage, it would be beneficial to include additional databases such as PubMed and Scopus. Furthermore, this study exclusively focuses on English-language literature, possibly overlooking valuable non-English-language literature. Additionally, the bibliometric analysis does not assess the quality of the searched literature. Lastly, the study only considers literature published between 2000 and 2023, indicating that researchers interested in the field should further analyze newly published research since 2024.

## Conclusions

Our analysis has shown a substantial increase in global research on frailty and HF, with the United States being at the forefront. Notably, Kentaro Kamiya from Kitasato University is one of the world’s prominent scientists in this field. Future research should focus on cardiac rehabilitation for HF patients with frailty, aiming to maintain their quality of life. Researchers can plan their studies by considering influential authors, journals, institutions, and citations to stay in line with emerging trends. Given the aging population, there is a pressing need for concerted efforts to decrease adverse cardiovascular events related to frailty. This can be accomplished by developing preventive strategies and primary prevention approaches, such as the early detection and management of frailty among the broader elderly population. Additionally, interventions tailored specifically for frail adults with HF should be developed.
